# Principles of Medical Practice

**Published:** 1881-02

**Authors:** W. W. Hall


### PRINCIPLES OF MEDICAL PRACTICE.

BY DR. W. W. HALL.

EVERY diseased condition arises from one great cause—an unequal circulation of the blood in the whole body, or in some part of it, at times no larger than the head of a pin; the severest torture of toothache and neuralgia is the result of the deranged condition of the circulation at some one point along the nerve of that part.

On the other hand, good health is the result of a natural, equable distribution of the blood to every portion of the system.

The highest effort of medical practice in the dispersion of disease and the recovery of health is directed toward equalizing the circulation; lessening it if too rapid, as in inflammation; hastening it if too slow, as in shivering or chills, and other forms of suffering. For example, if a part is burning with fever, it is because too much arterial blood is sent to that part, and with too great rapidity; hence we cut it off by gently bathing it with cold water, which invites the blood to a slower motion, and the man is well, for the fever has disappeared.

If the feet are cold it is because the blood is ceasing to circulate, is losing its life and heat; we put them in warm water, this heats the blood, invites a more vigorous flow, and soon all is well.

If you can bring warmth to a cool part, or cool a portion of the system which is in a fevered condition, you cure all the diseases which arise from too much cold or too much warmth.

Another condition of disease is where a very large amount of arterial or real blood is carried to a limited portion of the body, to the eye for example, or to the brain. We call this inflammation of the brain, and the man soon dies, unless relief is obtained; unless the eye is relieved, the sight is soon lost.

The most instantaneous method of relief in such cases is to place the patient in a chair, or set him up in bed, open a vein in the arm, and let the blood run until he is about to faint, and the man is safe for the hour, and proper treatment afterward establishes the safety, because the whole amount of blood in the body is diminished that much; the blood to each particular part is diminished that much, and by this diminution in the diseased part—diseased because there was too much blood—there is a return to the natural condition of healthful circulation; if actual faintness is induced the whole flow of blood to the diseased part is arrested, giving that part perfect relief and perfect rest for the time being. But suppose there is a diseased condition resulting to a part because there is too much blood in the veins instead of the arteries; then there is no redness, as in the arteries, because the blood of the veins is dark-colored; then the part is dark-colored, as in the result of a blow; or there is no blood, as in the paleness of cold; and instead of the sharp pain of rapidly moving arterial blood, there is the dull hurting of slow moving venous blood, so slow sometimes as almost to stop.

But whether the pain is sharp or dull, it is the result of too much blood being there; and in proportion as a remedy lessens the amount of blood by taking it away from the spot, out and out, as with leeches, or by inviting the excess of blood a short distance away to some point which, being in health, can better bear that excess, in such proportion do we give relief. A mustard plaster, for example, placed over the spot of an internal hurting, draws the blood to the surface, and relief is almost instantaneous.

But relieving pain and scattering disease and restoring health by the immediate extraction of blood from the part, although efficacious and instantaneous, is liable to abuse and to other objections; hence we come to the use of internal remedies, which are called drugs or medicines, accomplishing the same object, but in a more roundabout way; not so speedily, but more enduring, and with less harm in several directions.

As the point to be arrived at is to lessen the amount of blood in the diseased part, the only thing necessary to be done is to lessen the amount of fluids in the whole system, and this is to lessen the amount of blood, for all these fluids are made out of the blood; and to that extent the whole amount of blood is lessened as effectually as if by the lancet.

A boil gives intense suffering; lance it and relief is instantaneous; give a cathartic medicine in almost any bodily pain or suffering, as two or three tablespoons of castor-oil, or two or three tablespoons of Epsom salts, dissolved in a glass of water, and in a few hours most welcome relief is afforded. Sometimes a more speedy deliverance is effected by the use of an enema.

There is a gentler, safer and more permanent relief from bodily suffering, as a result of too much blood being in any particular part, but is not so expeditious; it is by giving medicines which are said to "act on the liver," stimulate it, make it work. This is because a large part of the blood of the body passes through the liver, and while it is in, its quantity is largely diminished by being used to make bile, which is passed directly into the bowels and out of the body ; hence a great deal more blood enters the liver than leaves it in the shape of blood. Thus any medicine, any remedy, which makes the liver work actively in thus making bile, diminishes the quantity of blood in the body, and gives relief.

But suppose the liver becomes so full of blood, its blood-channels clogged up, preventing any blood from passing along, then this large escape of fluid from the system is arrested, the evil of overfulness increases every moment, and every moment aggravating pain and intensifying the diseased condition.

The reader has now a complete idea of the principles of the practice of medicine in the treatment of disease and pain and suffering. It is narrowed down to the simple points of regulating the quality and quantity of the blood. If it is too thick, it will not run out—it clogs the machinery, it dams up the stream, arrests the flow, and that is death. If there is too much blood there is distention of the blood vessels ; they carry too much blood to a part, and disease and pain attack that part, to disorganize and destroy it effectually unless relief is obtained. But there is another way of lessening the amount of blood in the body, far better than any one, or all, mentioned above. Bleeding is depressive, and in other ways objectionable. The use of mustard plasters and setons and blisters is inconvenient and painful. To give violent carthartics, or even more moderate purgatives, is troublesome, nauseous and otherwise disagreeable. In most cases better headway can be obtained, and more human lives saved, if an attempt is made in all cases to diminish the amount of blood and equalize the circulation by DIET AND EXERCISE.

---

FROM the inquiries conducted by Professor Hermann Cohn, of Breslau, since 1865, it appears that short-sightedness is rarely or never born with those subject to it, and is almost always the result of strains sustained by the eye during study in early youth. Myopia, as it is called, is seldom found among pupils of village schools, and its frequency increases in proportion to the demand made upon the eye in higher schools and in colleges. A better construction of school desks, and an improved typography of textbooks, and a sufficient lighting of class-rooms are the remedies proposed to abate the malady.

